# Forced Feeding in Phthisis

**Published:** 1883-01

**Authors:** 


### FORCED FEEDING IN PHTHISIS.

THE practice known as forced feeding, or "super-alimentation," introduced by MM. Debové and Durjardin Beaumetz, Paris, has begun to attract some attention This super-alimentation consists in forcing into the patient's stomach, by means of a sound large quantities of highly concentrated food. It was first employed in phthisical patients who could not retain food on the stomach when taken in the ordinary way. Strange to say, these patients endured the unpleasantness of stomach-tubes, kept down the injected food, and improved in health. The method has now been extended to cases of hysteria with vomiting, also to dyspepsia and to various wasting diseases when the stomach is rebellious.

The food used is chiefly meat-powder, which is administered in milk, or bouillon, to which eggs may be added. This meat-powder appears to be a really useful preparation, and as it need not be expensive, it will doubtless become more widely employed. It is made by taking lean meat, mincing it, spreading the paste on porcelain tables and letting it dry at a temperature of 90°C. This is then taken and pounded into a powder, when it is ready for use. The dose given at first is small, being about twenty-five grammes.

at a meal. This amount is gradually increased until between four hundred and six hundred grammes are given daily. Such a dose is the equivalent of about four pounds of fresh meat. The amount of meat in an averaged diet is only about one pound, and when it is remembered that the meat-powder is administered dissolved in two liters of milk, to which several eggs are added, the significance of the term "super-alimentation" will be understood.

Under such a diet the urine is diminished in amount, but the urea is greatly increased, sometimes reaching eighty grammes per day—the normal amount being 32.5 grammes with an average diet. Sometimes albumen appears in the urine. Diarrhœa may occur, in which case pepsin and bismuth are added.

Phthisical patients rapidly gain weight, we are told, when thus forcibly fed, there being often an average daily increase of from eighty to one hundred grammes. Cough and expectoration diminish, and reparatory processes take place in the lungs. We do not hear it stated, however, to what extent permanent cures are produced.

It is hardly probable that American œsophagi will tamely submit to the introduction of a tube *ter in die*, and it is difficult to understand how such a process proved sedative to irritable stomachs, except in hysterical cases. The meat powders, and the over-feeding however are sufficiently rational measures in many cases.—*Medical Record.*

